# Mother root of *Aconitum carmichaelii Debeaux* exerts antinociceptive effect in Complet Freund’s Adjuvant-induced mice: roles of dynorpin/kappa-opioid system and transient receptor potential vanilloid type-1 ion channel

**DOI:** 10.1186/s12967-015-0636-4

**Published:** 2015-08-30

**Authors:** Chao Wang, Danni Sun, Chunfang Liu, Chunyan Zhu, Xianghong Jing, Shuping Chen, Cuiling Liu, Kai Zhi, Tengfei Xu, Hui Wang, Junling Liu, Ying Xu, Zhiqiang Liu, Na Lin

**Affiliations:** Institute of Chinese Materia Medica, China Academy of Chinese Medical Sciences, No. 16, Nanxiaojie, Dongzhimennei, Beijing, 100700 China; Institute Acupuncture and Moxibustion, China Academy of Chinese Medical Sciences, Beijing, 100700 China; National Center for Mass Spectrometry in Changchun and Jilin province Key Laboratory for Traditional Chinese Medicine Chemistry and Mass Spectrometry and Chemical Biology Laboratory, Changchun Institute of Applied Chemistry, Chinese Academy of Sciences, Changchun, 130022 China; University of Chinese Academy of Sciences, Beijing, 100049 China

**Keywords:** Processed mother root of *Aconitum carmichaelii*, CFA-induced inflammatory pain, Dynorpin/kappa-opioid system, TRPV1

## Abstract

**Background:**

Processed Chuanwu (PCW), the mother root of *Aconitum carmichaeliiDebeauxv*, has been widely used as a classic Traditional Chinese Medicine for pain relieve for over two millennia clinically. However, its action on chronic inflammatory pain has not been clarified. Here, we investigated the antinociceptive effect of PCW in complete freund’s adjuvant (CFA)-induced mice and its possible mechanisms associated with opioid system and TRPV1 ion channel.

**Methods:**

Male ICR mice were intraplantarly injected with CFA. PCW (0.34, 0.68 and 1.35 g/kg) was orally given to mice once a day for 7 days. Von frey hairs and planter test were assessed to evaluate the antinociceptive effect of PCW. To investigate the participation of dynorphin/opioid system in PCW antinociception, subtype-specific opioid receptor antagonists or anti-dynorphin A antiserum were used. To eliminate other central mechanisms that contribute to PCW antinociception, hot plate (50 °C) test were performed. Further, involvements of TRPV1 in PCW antinociception were evaluated in CFA-induced TRPV1^−/−^ and TRPV1^+/+^ C57BL/6 male mice, and in capsaicin-induced nociception ICR naive mice pretreated with nor-BNI. Meanwhile, calcium imaging was performed in HEK293T-TRPV1 cells. Finally, rotarod, open-field tests and body temperature measurement were carried out to assess side effects of PCW.

**Results:**

PCW dose-dependently attenuated mechanical and heat hypersensitivities with no tolerance, which could be partially attenuated by coadministration of k-opioid receptor antagonist nor-binaltorphimine (nor-BNI) or anti-dynorphin A (1–13) antiserum. And PCW antinociception was totally erased by pretreatment with nor-BNI in the hot plate test. In addition, PCW antinociception was decreased in TRPV1^−/−^ mice compared to TRPV1^+/+^ group. And PCW still manifested inhibitory effects in capsaicin-induced nociception with nor-BNI pretreatment. PCW significantly inhibited capsaicin-induced calcium influx in HEK293T-TRPV1 cells. Finally, no detectable side effects were found in naive mice treated with PCW.

**Conclusions:**

This study shows PCW’s potent antinociceptive effect in inflammatory conditions without obvious side effects. This effect may result from the activation of κ-opioid receptor via dynorpin release and the inhibition of TRPV1. These findings indicate that PCW might be a potential agent for the management of chronic inflammatory pain.

## Background

Inflammatory pain after tissue injury results in peripheral and central sensitization in peripheral tissues or spinal cord [1]. Indeed, hypersensitivities to thermal and mechanical stimuli are well documented characteristic symptom of chronic inflammatory pain [1, 2]. This chronic process causes patients to live with disability and continues to impart high health cost, economic loss to society. The mechanisms underlying the inflammatory pain remain to be elucidated. It is well-known that activation of opioid receptors result in the inhibition of chronic inflammatory pain [3]. On the other hand, transient receptor potential (TRP) ion channels in nociceptor peripheral terminals and dorsal root ganglias (DRG), especially TRPV1, contribute to the initiation and maintenance of hypersensitivity under inflammatory pain condition [4], and the deleting or inhibiting activity of TRPV1 leads to reduced inflammatory hyperalgesia [4–6]. Recently, several reports suggested that the activation of opioid receptors via inhibition of adenylyl cyclase suppresses TRPV1 and other nonselective cation currents stimulated by inflammatory agents to induce antinociception [7–9]. Current analgesics aimed to modulate pain transduction and transmission in neurons has limited success [1]. There are two main classes of analgesic drugs: opioids and non-steroidal anti-inflammatory drugs (NSAIDs), whereas opioid analgesics are limited by tolerance, somnolence, respiratory depression, confusion, constipation and addiction [10, 11], NSAIDs are restricted by side effects such as gastrointestinal ulcers, bleeding, myocardial infarction and stroke [3, 12, 13]. And the induction of hyperthermia by TRPV1 antagonists has hampered the development of new drugs as analgesics [14]. Therefore, novel and efficient agents that are lack of central side effects and of adverse effects typical of NSAIDs are needed [3].

There is a growing interest in the utilization of medicinal plants for prevention and treatment of pain. The mother and lateral root of *Aconitum carmichaelii Debx*, called as “Chuanwu” (CW) and “Fuzi” in Chinese respectively, have been widely used to relieve pain and treat rheumatic arthritis and other inflammatory conditions for over 2000 years in clinic. Previous studies have shown that processed Fuzi, obtained after autoclaving crude *Aconiti* tuber (*Ranunculaceae Aconitum carmichaeli Debeaux*; Iwate, Japan) to minimize its toxicity, are efficient in relieving pain in repeated cold stress mice, chronic constriction injury (CCI) neuropathic pain as well as adjuvant articular inflammation in rats [15, 16]. And the analgesic effect of processed Fuzi is mediated by spinal κ- and μ-opioid receptors in CCI rats and in tail-flick test [17–20]. On the other hand, it is found that CW has an anti-arthritic effect in complete Freund’s adjuvant (CFA)-induced arthritis rats [21], and methanol extracts of crude *Aconitum* roots have anti-inflammatory effects in inhibiting acid-induced vascular permeability and carrageen-induced hind paw edema in mice [22]. However, the action of processed CW (PCW) on chronic inflammatory pain remains unclear. In the present study, we investigated the antinociceptive effect of PCW in CFA-induced cutaneous inflammation and its possible mechanisms associated with opioid system and TRPV1 ion channel were also explored.

## Methods

This study was supported by the Research Ethics Committee of China Academy of Chinese Medical Sciences, Beijing, China (Permit Number: 2015–2028). All animals were treated in accordance with the guidelines and regulations for the use and care of animals at the Center for Laboratory Animal Care, China Academy of Chinese Medical Sciences and International Association for Suicide Prevention (IASP). All efforts were made to demonstrate consistent effects of the drug treatments and minimize the suffering of animals.

### PCW preparation and UPLC–MS and UPLC-MS^2^ analysis

#### PCW preparation

PCW was purchased from Beijing Huamiao Chinese medicine Engineering Development Center (Beijing, China) and authenticated by Professor Shilin Hu, China Academy of Chinese Medical Sciences. Hypaconitine and mesaconitine were purchased from the Chinese Authenticating Institute of Material and Biological Products (Beijing, China). Benzoylmesaconine, benzoylhypacoitine, aconitine, and benzoylaconitine were purchased from Lan Yuan Biological technology Co., Ltd. (Shanghai, China). Reserpine as the internal standard was purchased from Sigma (USA).

For PCW preparation, we used the method as we previously described [23, 24]. Briefly, PCW was dried, homogenized to fine powders by a plant pulverizer and screened by a 0.45 mm sieve. Then 50 g powdered PCW were immersed in 500 ml deionized water for 1 h, and heated to refluxing for 1.5 h. Water as 8 times of the above total weight was added for another 1.5 h refluxing after filtered. The filtered extraction solutions were concentrated to 50 ml, and kept at −20 °C and was diluted with deionized water to proper concentration for in vivo study. PCW extraction procedures were carried out according to our previous study [24].

#### UPLC-MS and UPLC-MS^2^ analysis

Chromatographic separation was performed on a Waters CORTECS UPLC BEH C18 Column (2.7 μm, 1.66 × 100 mm) keeping at 35 °C. 0.1 % aqueous formic acid (v/v) (A) and acetonitrile (B) were used as the mobile phase. The gradient elution with the flow rate of 0.3 mL/min was performed as follows: 10 % B at 0–2 min, 10–15 % B at 2–7 min, 15–30 % B at 7–15 min, 30–39 % B at 15–21 min, 39 % up to 100 % at 21–25 min. The sample inject volume was 5 μL. The MS analysis was carried out by the ESI source in both positive and negative ion mode, and full-scan mass range was 100–1,200 Da. The source temperature was 110 °C, and the desolvation gas temperature was 300 °C. The flow rates of cone and desolvation gas were set at 30 L/h and 600 L/h, respectively. The voltages of capillary, cone and extraction cone in positive ion mode were set at 2.5 kV, 35 V and 5.0 V, respectively, and in negative ion mode, they were set at 2.0 kV, 35 V and 5.0 V, respectively.

#### Data processing

Data were acquired with MassLynx 4.1 and processed for calibration and for quantification of the analytes with Target Lynx software (Micromass UK).

**Fig. 1:**
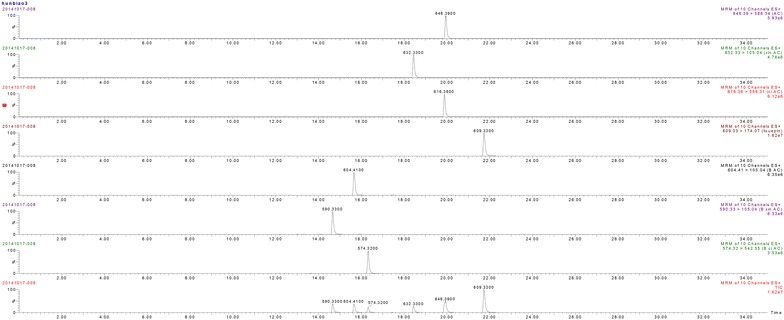
Typical multiple reaction monitoring chromatograms of PCW

Special conditions of MS/MS of each analyte are important for the development of a satisfactory quantification method by LC-MS/MS. Therefore, the intellistart function was used to find the most specific and sensitive detection parameters of each analyte in MRM mode. Ion transitions and instrumental parameters in MRM mode are shown in Table 1. Typical multiple reaction monitoring chromatograms of the compounds in positive ion mode are shown in Fig. 1.

**Table 1:** Ion transitions and instrumental parameters for their LC–MS/MS quantification in MRM mode

Sample	Ion mode	MRM	CV	CE	LOQ	LOD
Benzoylmesaconine	+	590.33 > 77.02	52	78	2.1	0.73
Aconitine	+	646.39 > 586.34	50	34	1.9	0.6
Mesantine	+	632.33 > 105.04	42	56	2.2	0.9
Benzoylhypacoitine	+	574.32 > 542.35	46	34	1.8	0.6
Benzoylaconitine	+	604.41 > 105.04	58	54	4.3	1.7
Hypaconitine	+	616.36 > 556.31	42	32	1.9	0.61
Reserpine(is)	+	609.33 > 174.07	40	42	–	–

*IS* internal standard, *CE* collision energy (eV), *CV* cone voltage (V), *MRM* specific mass transition, *LOQ* limit of quantification (ng/ml), *LOD* limit of detection (ng/ml)

The regression equations obtained by least squared regression using weighting factor (1/×2). Correlation coefficients (r2) were ≥0.9 for all calibration curves, and observed deviations were within ±15 % for all calibration concentrations. The calibration curves, calibration ranges and contented in PCW of these compounds are performed in Table 2.

**Table 2:** Calibration curves, concentration ranges and contented of 6 main components in PCW

Sample	Calibration curves	Concentration ranges (μg/ml)	Contented in PCW (μg/g)
Benzoylmesaconine	y = 0.6530*x + 0.0857 (r^2^ = 0.9991)	0.09–90	0.218
Aconitine	y = 0.5990*x + 0.0736 (r^2^ = 0.9875)	0.09–90	–
Benzoylhypacoitine	y = 0.3158*x + 0.0488 (r^2^ = 0.9928)	0.09–90	0.051
Benzoylaconitine	y = 0.9983*x + 0.0823 (r^2^ = 0.99665)	0.09–90	0.036
Hypaconitine	y = 0.5151*x + 0.0896 (r^2^ = 0.9778)	0.09–90	0.011
Mesactonine	y = 0.4919*x + 0.0520 (r^2^ = 0.9903)	0.09–90	0.006

### Animals

Male mice of 8–12 weeks old were used in all experiments. C57BL/6 TRPV1-knockout (KO) mice (TRPV1^−/−^) were purchased from Jackson Lab (USA). ICR mice and C57BL/6 wild-type (WT) mice were purchased from Laboratory Animal Center of Academy of Military Medical Sciences, Beijing, China (License No. SCXK-2012-004). They were kept in a temperature controlled environment (22 ± 1 °C), 55 ± 5 % relative humidity with a 12h:12 h light–dark cycle and fed with standard chow, for at least 1 week before any manipulations. Animals were habituated to the laboratory conditions for at least 1 h before testing and all experiments were performed during the light phase of the cycle. All the experiments were performed by two independent, blinded observers.

### CFA-induced chronic inflammatory pain

ICR mice were randomly divided into six groups (n = 8): the control group (Control), the CFA (Sigma-Aldrich, St.Louis, MO, USA)-induced inflammatory group (CFA), CFA-induced mice treated with PCW groups (CFA + PCW 0.34, 0.68, and 1.35 g/kg, respectively), and control mice treated with PCW group (PCW 1.35 g/kg). CFA or control groups received an intraplantar injection of 20 μl of CFA or vehicle (2 % tween 80 plus saline) to the right hind paw. 48 h later, mice were orally administrated by syringe feeding with distilled water (10 ml/kg) or PCW daily for 7 days and mechanical and thermal hypersensitivities were evaluated accordingly.

#### Measurement of mechanical hypersensitivity

Mice were acclimatized in individual clear boxes on wire-mesh plat form, mechanical hypersensitivity was assessed by the sensitivity to the application of von Frey hairs (Stoelting Co., Chicago, USA). The von Frey filaments of 0.04–2.0 g were used and held perpendicularly to the plantar surface of the injected paw for 2–4 s. An abrupt withdrawal of injured paw or flinching behaviour immediately following removal of von Frey hairs indicate positive responses. A 50 % paw withdrawal threshold (PWT) was calculated following Dixon’s up-down method [25]. The PWT was analyzed at 0, 0.5, 1, 1.5, 2, 3, 4, 24 h and 3rd, 5th, 7th days post PCW administration, respectively.

#### Measurement of the heat hypersensitivity in plantar test

The plantar test was assessed for thermal hypersensitivity as previously described [26]. Briefly, mice were acclimatized to an apparatus (Ugo Basile Srl, Comerio VA, Italy) consisting of individual Perspex boxes, an infrared radiant heat source was directed to middle part of the plantar surface of the hind paw. We adjusted the basal paw withdrawal latency (PWL) to 9–12 s and set a cut-off time of 20 s to prevent tissue damage. PWL were measured at time piont of 1 h on the 2nd, 4th, 6th day post PCW (0.34–1.35 g/kg, p.o.) or vehicle administration, using the same mouse that for mechanical hypersensitivity assessment, and three trials for each hind paw. The interval of PWL measurement was 5 min.

### Investigation the mechanisms of antinociception of PCW in mice

#### Involvement of opiod system

To determine the potential role of opiod system in the antinociceptive effects of PCW, CFA-induced mice were pretreated with cyprodime (2.3 μmol/kg, i.p., a selective μ-opioid receptor antagonist, Sigma) or nor-binaltorphimine (nor-BNI, 13.6 μmol/kg, s.c., a preferential κ-opioid receptor antagonist, Sigma) or naltrindole (11.1 μmol/kg, i.p., a selective δ-opioid receptor antagonist, Sigma). 15 min later, animals received an administration of PCW (1.35 g/kg, p.o.) or distilled water (10 ml/kg, p.o.), and PWT of injured paw was evaluated 1 h later. In another set of experiments, mice co-treated with PCW and nor-BNI were subjected to the plantar test for heat hypersensitivity as described above.

Since endogenous dynorphin plays an important role in nociception modulation in inflammatory conditions [27], we evaluate the role of endogenous dynorphin in PCW antinociception by intraspinal injection (i.t.) of anti-dynorphin A (1–13) antiserum (Bachem/Peninsula Laboratories, Belmont, CA, USA) in CFA-induced mice. 72 h after CFA treatment, mice were received a dose of anti-dynorphin A (1–13) antiserum (5 μg/5 μl, i.t.), then, PCW (1.35 g/kg, p.o.) was immediately administrated, and mechanical or heat hypersensitivities were measured 1 h later. The anti-dynorphin A (1–13) antiserum was dissolved in saline and was injected by a 29-gauge needle between the L5 and L6 intervertebral spaces within 5 min [28].

To explore whether other possible central mechanisms contribute to PCW antinociceptive effect, heat hypersensitivity to hot plate was evaluated as previously described [29]. Briefly, CFA-induced mice were pretreated with nor-BNI (13.6 μmol/kg, s.c.). 15 min later, animals received an administration of PCW (1.35 g/kg, p.o.) or distilled water (10 ml/kg, p.o.), then mice were placed in a hot plate set at 50 ± 1 °C (Cold-hot Plate, Ugo Basile, Comerio, Italy) and the nociception was recorded as the latency time to withdrawal, shaking or licking the injured paw 1 h later. A cut-off time of 20 s was set to avoid tissue damage.

#### Involvement of TRPV1 ion channel

To evaluate the possible involvement of TRPV1 ion channel on PCW antinociceptive effect, TRPV1^+/+^ and TRPV1^−/−^ mice were used. First, baseline sensory thresholds in TRPV1^+/+^ and TRPV1^−/−^ mice were tested, then mice received an intraplantar injection of 20 μl of CFA to the right hind paw. 48 h later, mice were orally administrated by syringe feeding with PCW (1.35 g/kg) or distilled water once. PWT of the injured paw was assessed before and 0.5, 1, 2, 3, 4 h post-PCW or distilled water administration, respectively.

To further explore the possible involvement of TRPV1 in PCW antinociception, capsaicin-induced spontaneous nociception, mechanical and thermal hypersensitivities were assessed pretreated with κ-opioid receptor antagonist, as previously described [30, 31]. Mice were pretreated with AMG9810 (a selective TRPV1 antagonist, 30 mg/kg, i.p., Tocris Bioscience, Ellisville, Missouri, USA) or Nor-BNI (13.6 μmol/kg, s.c., 15 min before PCW administration), PCW (1.35 g/kg, p.o.) or distilled water (10 ml/kg, p.o.) 1 h (for p.o. administration) or 0.5 h (for i.p. administration) prior to the injection of 20 µl capsaicin (2 µg/paw, Tocris Bioscience) or vehicle to the plantar surface of the right hind paw, respectively. Immediately after capsaicin application, mice were placed into clear observation boxes and the nociceptive response was evaluated as the time of spent licking the injected paw during 5 min. In another set of experiments, the same mice were immediately put into the individual clear boxes on wire-mesh platform for PWT assessment 15 min after capsaicin administration. In the third experiment, mice received the same administrations of AMG9810, Nor-BNI, PCW or distilled water, as described above, and then mice were acclimatized in individual clear boxes for 1 h. Immediately after vehicle or capsaicin intraplantar injection, mice were again put into the individual clear boxes, heat hypersensitivity of the injured paw was assessed in planter test, 15 min after capsaicin treatment.

### Side effects assessments

#### Rotarod and open-field test

The effects of PCW on locomotor activity was assessed as previously reported [32]. Briefly, mice were trained on the rotarod (8 rpm) until they could remain on the apparatus for 1 min without falling. Then, mice were subjected to rotarod test 1 h after PCW (1.35 g/kg, p.o.) or vehicle (10 ml/kg) administration. The number of falls and latency to first fall from the apparatus were recorded for duration of 4 min. To exclude possible nonspecific muscle relaxant or sedative effects, mice were subjected to the open-field test [32]. The floor of the arena was divided into 12 equal squares, and the number of squares crossed with all paws was counted in a 5-min session.

#### Body temperature

The difference between the values before and after PCW (1.35 g/kg, p.o.) administration were calculated (Δ °C) as described previously [32]. The TRPV1 antagonist (AMG9810) (30 μmol/kg, p.o.) was used as a positive control.

### Cell viability assay

Human embryonic kidney (HEK293) cells were seeded in 96-well plates and incubated in serum free sterile DMEM supplemented with 100 U/mL penicillin, 100 μg/mL streptomycin, and 2 mM Gln-glutamine for 24 h. Cells were then incubated with medium containing (0.25, 0.5, 1 µg/ml, respectively) for 24 h. After treatment, cells were washed twice with phosphate-buffered saline (PBS, pH 7.4), and then cell viability was determined by 3-(4,5-Dimethylthiazol-2-yl)-2,5-diphenyltetrazolium bromide (MTT) method using Cell Titer 96^®^ Non-Radioactive Cell Proliferation Assay (Promega, Madison, USA) according to the manufacturer’s instructions. All absorbances at 570 nm were measured with a Tecan Infinite M200 Pro microplate reader (Tecan, Mannedorf, Swizerland). The experiments were carried out 2 times in triplicate measurements.

### Calcium imaging

#### Cloning and cell culture

TRPV1 sequence was amplified from homo genome and cloned into pMig plasmid (Addgene, #9044). HEK293T cells were cultured in DMEM with 10 % fetal calf serum, 100 U/l penicillin and 100 μg/ml streptomycin at 37 °C, 5 % CO_2_. Cells seeded on poly-l-lysine-covered coverslips at the density of 1 × 10^6^/6-well plate (four coverslips/one well of 6-well plate) were transfected with 4 μg pMig-TRPV1 by lipofectamine^®^ 2000 (Invitrogen, Mount Waverley, Australia) and used for functional calcium imaging within 24–48 h after transfection.

#### Detection of intracellular Ca^2+^

Cells plated on coverslips in 6-well plated were transfected and cultured in calcium-containing DMEM overnight and then treated with PCW (0.25, 0.5, 1 µg/ml, respectively) for 30 min. Fluo-4AM (40 µM) was loaded into HEK293T-TRPV1 cells for 30 min at 37 °C and then 30 min at room temperature. Cells were then treated with capsaicin (8 μM). Fluorescence changes were examined by a Zeiss Lsm710 confocal microscope (Carl Zeiss AG, Oberkochen, Germany). The experiments were repeated independently for two times in triplicate measurements.

### Statistical analysis

Data are presented as mean ± SEM. Data obtained from mechanical and thermal hypersensitivity experiments were analyzed by two-way ANOVA followed by Bonferroni post hoc test. Other data were analyzed by one-way analysis of variance (ANOVA) followed by Student–Newman–Keuls post hoc test. A value of *P* < 0.05 was taken to be significant.

## Results

### PCW attenuated mechanical and heat hypersensitivities in CFA-induced mice

**Fig. 2:**
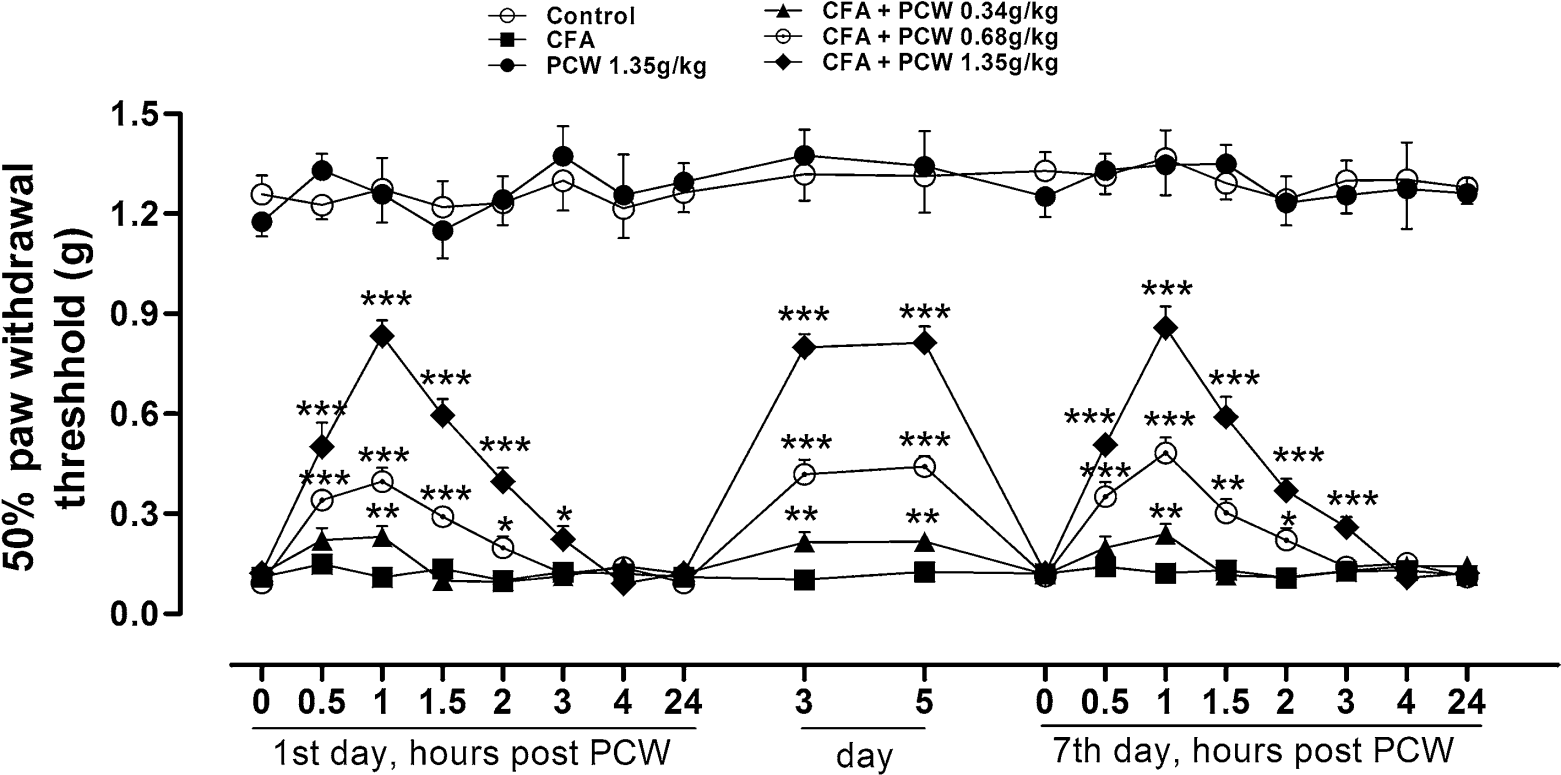
Effect of PCW on mechanical hypersensitivity induced by CFA in mice. CFA-induced inflammatory pain mice were orally administrated with PCW (0.34, 0.68, and 1.35 g/kg, respectively) or water daily for 7 days. On the 1st and 7th days, evaluations for mechanical hypersensitivity were done 0, 0.5, 1, 1.5, 2, 3, 4 and 24 h post-PCW treatment; all other evaluations were done 1 h post-treatment. Data are represented as the mean ± SEM. (n = 8). *P<0.05, **P<0.01 and ***P<0.001 vs. the CFA group, respectively

To investigate the antinociceptive effects of PCW, a chronic inflammatory model of nociception was used. Mechanical hypersensitivity was evaluated from day 1 to day 7 48 h after an intraplantar injection of CFA in mice. As showed in Fig. 2, CFA caused significant mechanical hypersensitivity characterized by the reduced PWT compared with control group. Oral administration of PCW (0.34, 0.68, and 1.35 g/kg) was able to significantly reverse mechanical hypersensitivity and this effect lasted up to 3 h in high dose group. The maximum effect was observed 1 h post PCW treatment. And this antinociceptive effect was maintained while PCW (0.34, 0.68, and 1.35 g/kg) was orally administered daily, until the 7th day post-treatment. Notably, on the 7th day, PCW reduced mechanical hypersensitivity with a time-course effect profile similar to that of the first day, ruling out the possibility of drug tolerance. Although PCW (1.35 g/kg) potently reduced mechanical hypersensitivity in CFA-induced nociception, it did not alter the baseline thresholds in normal control mice, suggesting that PCW (0.34–1.35 g/kg, p.o.) has a unique role in the normalization of inflammatory pain.

**Fig. 3:**
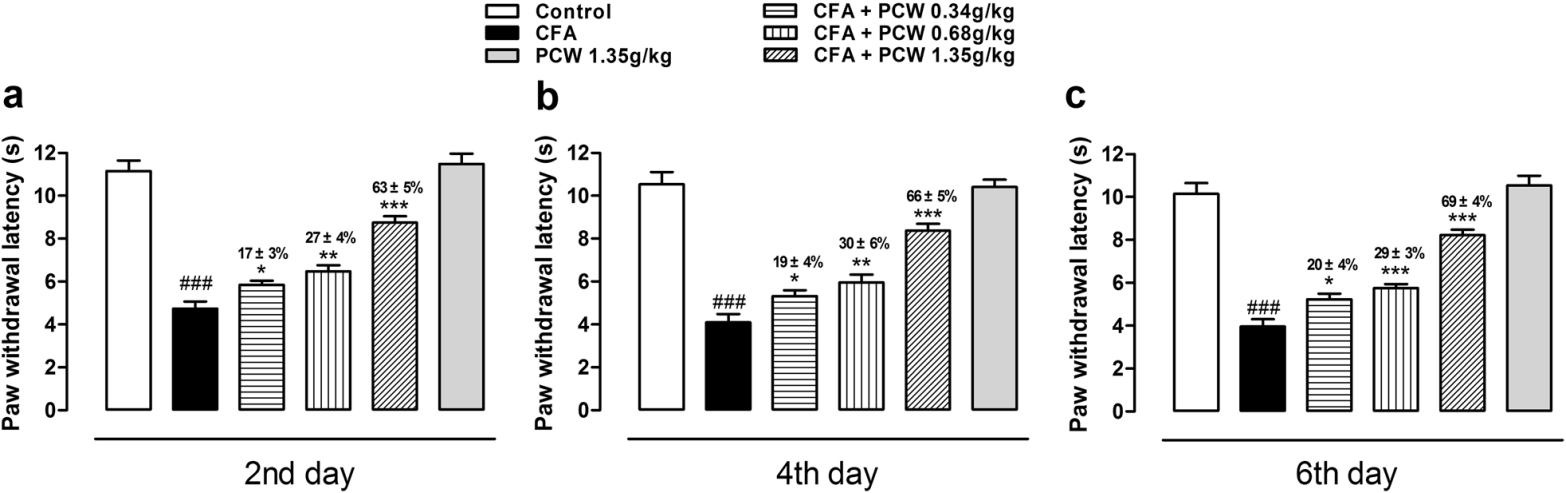
Effect of PCW on CFA-induced heat hypersensitivity in mice. Evaluations of heat hypersensitivity were done 1 h post-treatment in a plantar test on the 2nd (**a**), 4th (**b**) and 6th (**c**) days using the same mice for mechanical hypersensitivity assessment. Data are represented as the mean ± SEM. (n = 8). And ^###^ *P* < 0.001 vs. the control group; *P < 0.05, **P < 0.01 and ***P < 0.001 vs. the CFA group, respectively

Hypersensitivity to heat stimulus was explored in CFA-induced mice in a plantar test. As showed in Fig. 3, oral administration of PCW significantly reversed heat hypersensitivity in a dose-dependent manner. And similar inhibitions were observed in each dose of PCW on the 2nd, 4th and 6th days after treatment. These results also suggested that the antinociceptive effects of PCW were not susceptible to tolerance. Consistent with mechanical results above, PCW (1.35 g/kg) did not produce any analgesic effect per se in a plantar test even with repeated administration of 7 consetutive days.

### Involvement of kappa-opioid/dynorphin system in PCW antinociception in CFA-induced mice

**Fig. 4:**
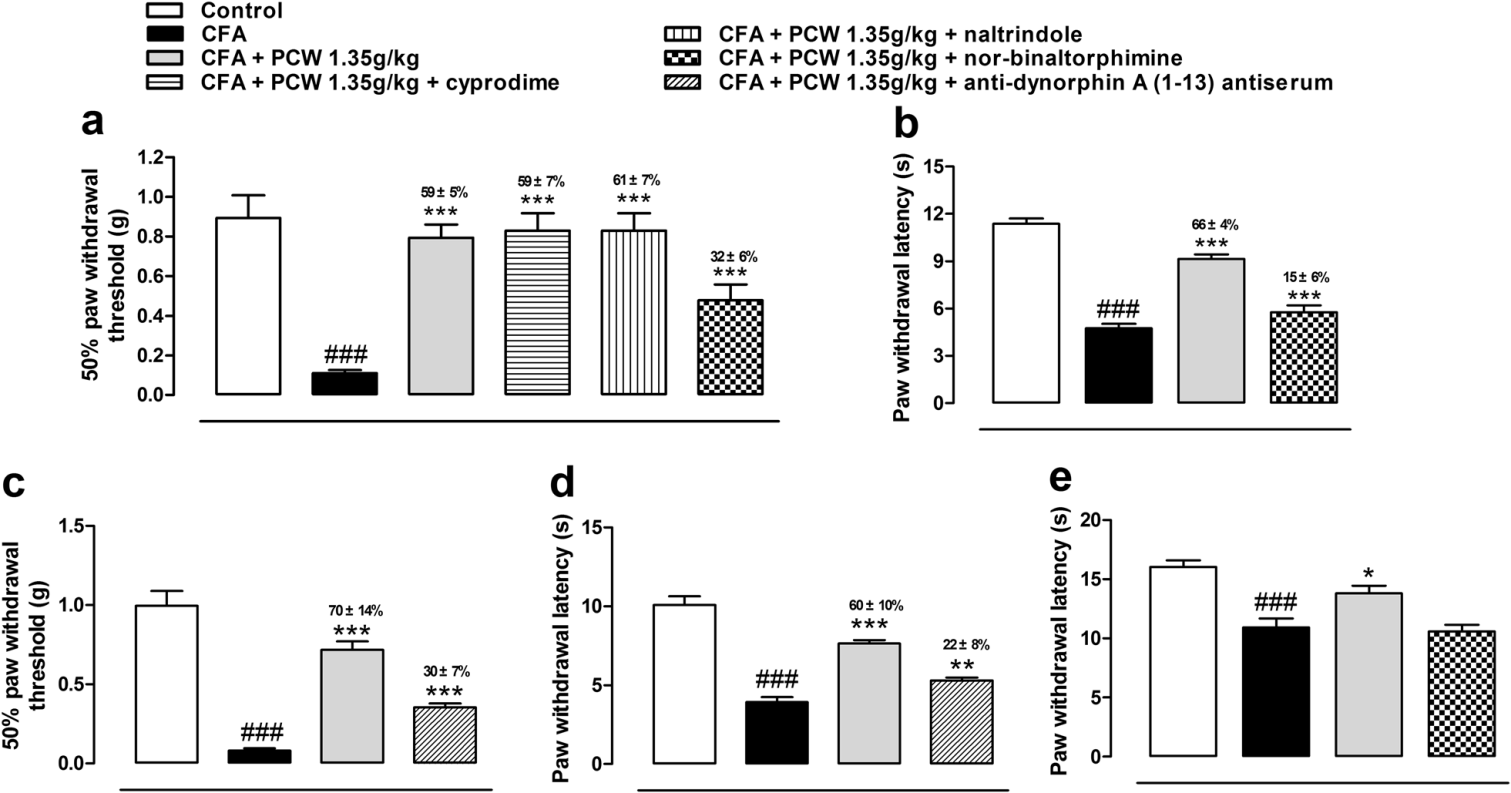
Involvement of opiod/dynorphin system in the antinociceptive effect of PCW in CFA-induced mice. **a** Mice were intraplatar injected with CFA, 48 h later, mice were pretreated with cyprodime (2.3 μmol/kg, i.p.) or nor-binaltorphimine (nor-BNI, 13.6 μmol/kg, s.c.) or naltrindole (11.1 μmol/kg, i.p.). After 15 min, the mice received an administration of PCW (1.35 g/kg, p.o.) or distilled water (10 ml/kg, p.o.), and the mechanical hypersensitivity of right hind paw was evaluated 1 h later. Antinociceptive effects of PCW were also evaluated co-administration of nor-BNI (**a**, **b**) and anti-dynorphin A (1–13) antiserum (**c**, **d**) in mechanical and heat hypersensitivity tests, and this effect was also performed pretreated with nor-BNI in the hot plate test (**e**). Data are represented as the mean ± SEM. (n = 6). ^###^ *P* < 0.001 vs. the control group; *P < 0.05 and ***P < 0.001 vs. the CFA group, respectively

Previous reported that activations of μ-, δ-, and κ-opioid receptors lead to significant reduced inflammatory hypersensitivities [33–36]. To assess the antinociceptive mechanism of PCW on opioid system, specific antagonists of opioid receptors were used. As showed in Fig. 4a, PWT was strikingly reduced 48 h post CFA treatment. PCW (1.35 g/kg) significantly reversed PWT with inhibition of 59 ± 5 %, and applications of μ- or δ-opioid receptor antagonist cyprodime or naltrindole did not alter the antinociceptive effect of PCW with similar inhibitions of 59 ± 7 and 61 ± 7 %, respectively. Interestingly, when nor-BNI, a selective antagonist of κ-opioid receptor, was used, the antinociceptive effects of PCW significantly reduced, with an inhibition of 32 ± 6 %. Heat hypersensitivity in a plantar test was also examined. As demonstrated in Fig. 4b, CFA caused a significant reduced in heat hypersensitivity, and this effect were reversed by treatment of PCW with inhibition of 66 ± 4 %, while nor-BNI could significantly reverse its antinociception with an inhibition of 15 ± 6 %. These results indicated that the activation of κ-opioid receptor, but not μ- or δ-opioid receptor, contribute to the antinociceptive effect of PCW.

Endogenous dynorphin that binds to and activates κ-opioid receptor plays important role in pain modulation under inflammtory conditons [27]. We further investigated whether PCW exerted its antinociceptive effects via dynorphin release in the spinal cord. As demonstrated in Fig. 4c, d, PCW significantly increased PWT and PWL compared to CFA group, and these effects could be significantly decreased by coadministration of anti-dynorphin A (1–13) antiserum (i.t.), with similar inhibition as in the nor-BNI-treared mice described above.

To explore whether other possible central mechanisms contribute to the antinociceptive effects of PCW, heat hypersensitivity in a hot plate test was further investigated. As showed in Fig. 4e, CFA obviously reduced latency time to paw withdrawal compared to control group, and PCW (1.35 g/kg, p.o.) could reverse the latency time, while this effect was totally blocked by nor-BNI pretreatment, even in a higher dose of PCW (3.00 g/kg, p.o.) (data not shown). These results supported the central analgesic mechanism of PCW that the activation of κ-opioid receptor contributes to its antinociceptive process, and it also suggested that other central mechanisms may not be involved in the antinociceptive effects of PCW under inflammatory conditions.

### Involvement of TRPV1 in PCW antinociception

**Fig. 5:**
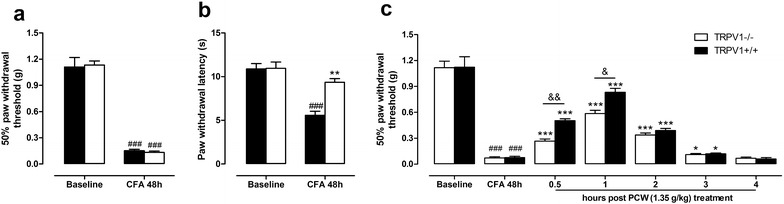
Antinociceptive effects of PCW in CFA-induced TRPV1^−/−^ and TRPV1^+/+^ mice. Baselines of PWT and PWL were tested in TRPV1^−/−^ and TRPV1^+/+^ mice before and 48 h after CFA treatment in the right hind paw (**a**) and (**b**) (n = 6). Then, mice were orally administrated by syringe feeding with PCW (1.35 g/kg) or vehicle (10 ml/kg) once. Mechanical hypersensitivity of the injured paw was assessed by the sensitivity to the application of von Frey hairs 0, 0.5, 1, 2, 3 h, post-PCW administration, respectively (**c**) (n = 8). Data are represented as the mean ± SEM. (n = 6). ^###^ *P* < 0.001 vs. the baseline; *P < 0.05 and ***P < 0.001 vs. the CFA group, respectively; ^&^ *P* < 0.05 and ^&&^ *P* < 0.01 indicate TRPV1^−/−^ vs. the TRPV1^+/+^ group, respectively

TRPV1 ion channel plays an important role in the initiation and maintenance of mechanical and heat hypersensitivities in inflammatory conditions [4–6]. We investigated whether TRPV1 was involved in the antinociceptive effect of PCW. As demonstrated in Fig. 5a, 48 h after CFA injection, both TRPV1^+/+^ and TRPV1^−/−^ mice exhibited decreased PWT. By contrast, CFA reduced the PWL in wild-type mice, but not in TRPV1^−/−^ group (Fig. 5b). Therefore, PWT were chosen for the assessments of TRPV1 ion channel in the antinociceptive effects of PCW. As showed in Fig. 5c, PCW caused a rebound of mechanical sensitivity toward baselines both in TRPV1^+/+^ and TRPV1^−/−^ mice 0.5, 1 and 2 h post treatment, and the inhibitions in TRPV1^+/+^ mice were about 23 % higher than that in TRPV1^−/−^ group at 0.5 and 1 h post PCW treatment. These data suggested that TRPV1 relate to the antinociceptive effect of PCW.

**Fig. 6:**
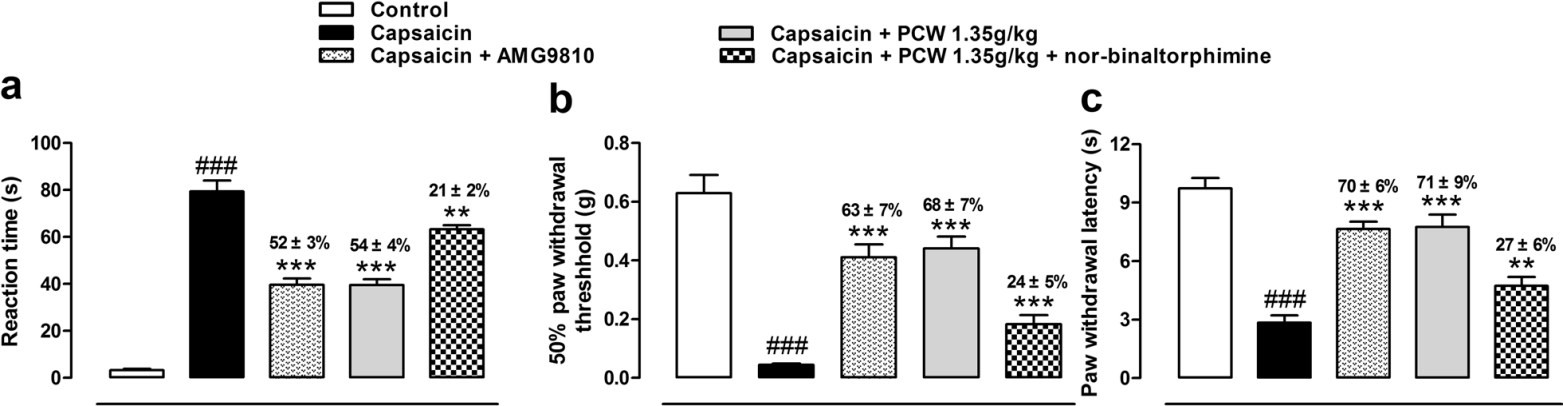
Antinociceptive effects of PCW co-treated with Nor-BNI in capsaicin tests. Nor-BNI (13.6 μmol/kg, s.c., 15 min before) or AMG9810 (30 mg/kg, i.p., 30 min before) were pretreated to mice. Then PCW (1.35 g/kg, p.o.) or distilled water (10 ml/kg, p.o.) were administered, and capsaicin tests were performed 0.5 (for i.p. administration) or 1 h (for p.o. administration) later. Immediately after capsaicin injection, the nociceptive responses were evaluated as the time spent licking the injected paw during 5 min (**a**), and the mechanical (15 min after capsaicin injection) (**b**), thermal (15 min after capsaicin injection) (**c**) hypersensitivities were also assessed. Data are represented as the mean ± SEM. (n = 6). ^###^ *P* < 0.001 vs. the control group; **P < 0.01 and ***P < 0.001 vs. the capsaicin group, respectively

To get the knowledge on the antinociceptive effects of PCW through TRPV1 ion channel, capsaicin-induced nociception was further examined. As shown in Fig. 6a, capsaicin, a specific activator of TRPV1, induced obvious spontaneous nociception, and this nociception was significantly reduced in AMG9810 and PCW-treated groups with similar inhibitions of 52 ± 3 and 54 ± 4 %, respectively. While pretreated with nor-BNI, PCW produced an inhibition of 21 ± 2 %. Capsaicin also induced obvious mechanical and thermal hypersensitivities, which were reduced by AMG9810 and PCW with similar inhibitions, and PCW could reverse these hypersensitivities with pretreatment of nor-BNI with inhibitions of 24 ± 5 and 27 ± 6 %, respectively (Fig. 6b, c). These results further supported the antinociceptive effects of PCW in a capaicin-induced nociception test, and this antinociception was also independent of the activation of κ-opioid receptor.

**Fig. 7:**
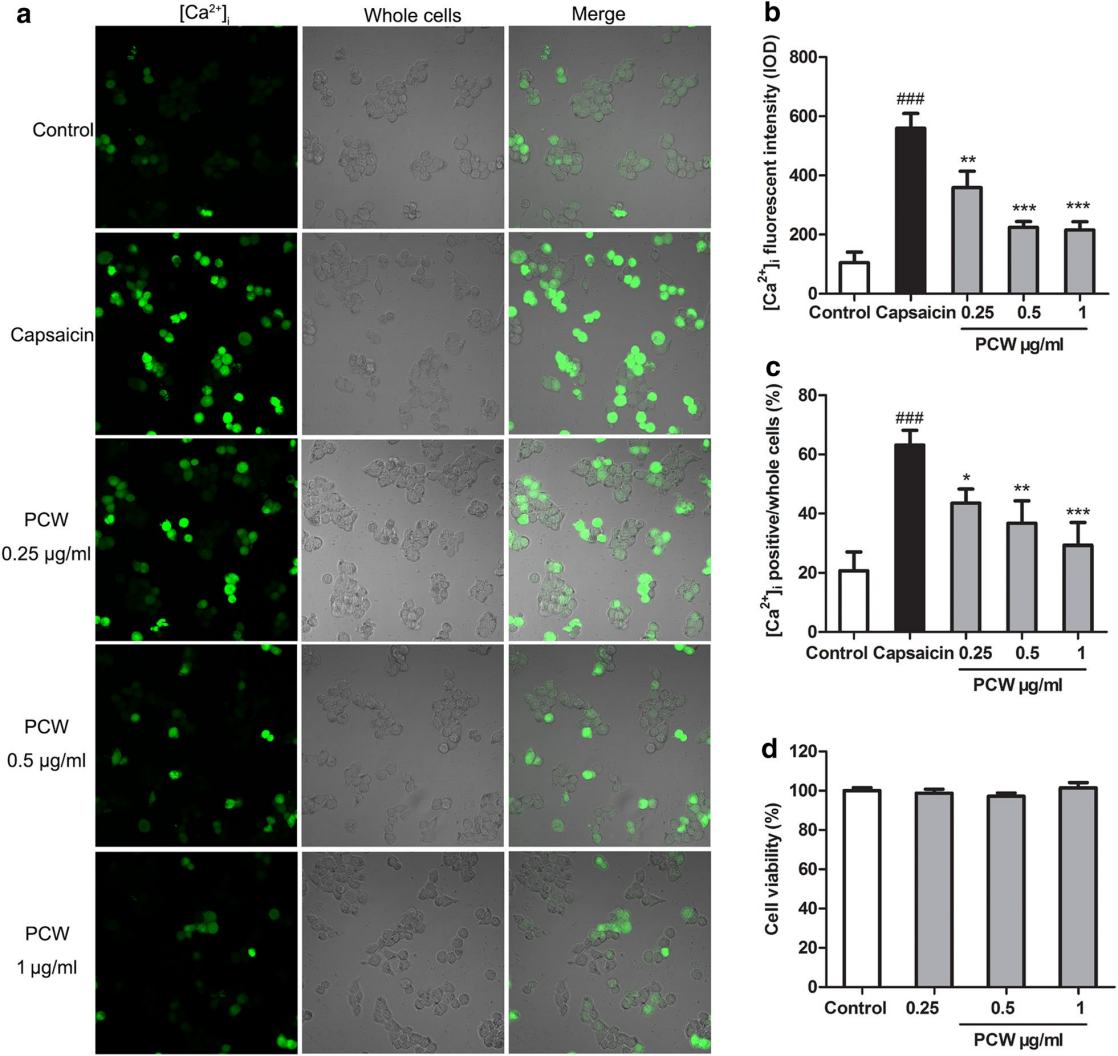
Effect of PCW on capsaicin-induced [Ca^2+^]_i_ influx in HEK293T-RPV1 cells. HEK293T-RPV1 cells were incubated with PCW (0.25, 0.5 and 1 μg/ml, respectively) for 30 min, then Fluo-4AM for 1 h, and immediately stimulated with or without capsaicin (8 µM). The cells were divided into five groups: Control–HEK293T-TRPV1 cultured cells; Capsaicin–capsaicin-induced cells; PCW groups–capsaicin-induced cells treated with various concentrations of PCW (0.25, 0.5 and 1 μg/ml, respectively). **a** Localization of [Ca^2+^]_i_ (*green*) in HEK293T-RPV1 cells with by fluorescence staining and laser scanning microscopy. **b** Fluorescent intensity of [Ca^2+^]_i_ in HEK293T-RPV1 cells. **c** Mean change in fluorescence ratio in HEK293T-RPV1 cells. 10 microscopic fields were selected randomly and [Ca^2+^]_i_ positive cells were counted. **d** No effect of PCW (0.25, 0.5 and 1 μg/ml, respectively) on the cell viability by 3-(4,5-Dimethylthiazol-2-yl)-2,5-diphenyltetrazolium bromide method. Cell viability of the control was taken as 100 %. Data are represented as the mean ± SEM. ^###^ *P* < 0.001 vs. the control group; *P < 0.05, **P < 0.01 and ***P < 0.001 vs. the capsaicin group, respectively. n = 3 in each group and each assay was repeated 2 times

To further explore the effect of PCW on TRPV1 activity, the intracellular calcium levels were measured in HEK293T-TRPV1 cells. As demonstrated in Fig. 7a–c, capsaicin significantly increased intracellular calcium in HEK293T-TRPV1 cells, while PCW (0.25–1 µg/ml) dose-dependently decreased capsaicin-induced calcium influx. These results suggested that PCW has direct inhibitory effect on TRPV1 activity. Next, we examined whether the above effect was due to its cytotoxicity. Our results showed that PCW (0.25–1 µg/ml) did not exert any cytotoxic effects on HEK293T cells under the experimental conditions used in the present study (Fig. 7d), suggesting that PCW might specifically inhibit TRPV1 activity in vitro.

### PCW did not induce detectable adverse effects

Since PCW may stimulate κ-opioid receptor via dynorphin release to produce antinociception, we investigated whether this medicine produced common side effects of opioid-like drugs in rodents on motor performance. The active dose of PCW (1.35 g/kg, p.o.) did not alter forced or spontaneous locomotion, as assessed by the rotarod and open-field tests, respectively (Table 3). Meanwhile, PCW may inhibit TRPV1 activity to produce antinociception. Thus, we further explored whether PCW caused body temperature alteration as TRPV1 antagonists [14]. As demonstrated in Table 3, PCW (1.35 g/kg p.o.) did not induced hyperthermia compared to vehicle group, while the TRPV1 antagonist AMG9810 induced a significant increase in rectal temperature. In addition, PCW treatment for 7 days did not induce significant body weight change and ulceration of the gastric mucosa compared with CFA and normal control groups (data not shown).

**Table 3:** The effects of PCW on locomotion and body temprature of mice 1 h after administration

Treatment	Rotarod test		Open-field test		Body temperature
	First fall (s)	Falls	Crossing	Rearing	
Vehicle	176 ± 21	2.3 ± 0.5	47 ± 8	11 ± 1	0.29 ± 0.06
PCW	183 ± 18	1.7 ± 0.3	54 ± 6	9 ± 1	0.36 ± 0.06
AMG9810	–	–	–	–	1.09 ± 0.12***

Mice were randomly divided into 3 groups: the Vehicle (10 ml/kg, p.o.) group, PCW (1.35 g/kg, p.o.) group and AMG9810 (30 μmol/kg, p.o.)-treated group for rotarod test, open-field test or body temperature assessment. Significant differences were not observed in the locomotion or body temprature tests between vehicle and PCW (1.34 g/kg. p.o.) groups, while AMG9810 (30 μmol/kg, p.o.) significantly increased rectal temperature compared to vehicle or PCW (1.34 g/kg. p.o.) groups. Data are expressed as mean ± SEM. (n = 6)

*** P < 0.001 vs. the vehicle or PCW groups

## Discussion

PCW is known to be used effectively to treat joint pain and inflammatory diseases in clinic. However, its antinociceptive properties and the possible mechanisms remain unclear. Data presented in this study indicate PCW could potently attenuate hypersensitivities to mechanical and heat stimuli without tolerance in CFA-induced nociception. Moreover, the antinociception of PCW was partially reversed by coadministration with nor-BNI and anti-dynorphin A antiserum, respectively. And this antinociceptive effect was also reduced in TRPV1^−/−^ mice. In addition, PCW could effectively inhibit capsaicin-induced nociceptive behaviors with coadministration of nor-BNI. PCW also reduced capsaicin-induced calcium influx in HEK293T-TRPV1 cells. Thus, our data showed the potent antinociceptive effects of PCW and pointed to its connection with dynorphin/κ-opioid system and TRPV1 in inflammatory conditions.

CFA-induced cutaneous inflammation in rodents effectively mimics a chronic inflammatory pain condition. A characteristic symptom of this model is that it displays hyperalgesia to mechanical and thermal stimulation [1, 2], and has been widely used to study persistent inflammatory pain. In this study, we analyzed the antinociceptive effects of PCW in CFA-induced cutaneous inflammation. Our results showed that oral administration of PCW at doses of 0.34–1.35 g/kg could remarkably reduce inflammatory mechanical and heat hypersensitivities without tolerance, which confirmed its antinociceptive action in clinical use for pain relieve.

Recent studies reported that opioid drugs lead to more-pronounced antinociceptive effects on inflammatory pain [33–36]. Previous reports suggested that κ- and μ-opioid receptors are involved in the antinociceptive effects of processed Fuzi (the lateral root of *Aconitum carmichaelii Debx.*) [17–20]. These studies encourage us to screen the specific opioid receptors that relate to PCW antinociception by using selective opioid receptors antagonists. Our results showed that pretreatment with nor-binaltorphimine, a preferential κ-opioid receptor antagonist, could markedly eliminate the antinociception of PCW, while treatments of cyprodime, a selective μ-opioid receptor antagonist and naltrindole, a selective δ-opioid receptor antagonist did not reduce the analgesic effects of PCW, indicating that κ-opioid receptor, but not μ-, or δ-opioid receptor contributes to the antinociceptive effect of PCW. It has been reported that endogenous dynorphin synthsis increased in the lumber spinal cord and plays an important role in nociception modulation under inflammatory conditions [27]. In the present study, the enhanced antinociceptive effect of PCW under inflammatory conditions may result from the increased release of endogenous dynorphin from both PCW application and CFA-induced nociception in the spinal cord and the subsequent activation of kappa opiod receptor. And this hypothesis was supported by our findings that the antinociceptive effects of PCW significantly dcreased by anti-dynorphin A (1–13) antiserum coadministration (i.t.). Although the antinociception of PCW in CFA-induced inflammation still remained with coadministration of nor-BNI or anti-dynorphin A (1–13) antiserum, PCW did not produce any antinociception with coadministration of nor-BNI in the hot plate test, suggesting that the antinociceptive effects of PCW may also relate to certain peripheral mechanisms.

TRPV1 is a heat transducer in normal and pathological conditions, activated by temperatures above 42 °C, and deleting or inhibiting the activity of this heat-sensitive channel results in reducing inflammatory heat and mechanical hyperalgesia [4–6]. Considering the close connections of this ion channel with mechanical and heat hypersensitivities in inflammatory pain conditions, it is reasonable to think that the antinociceptive effect of PCW may be mediated by TRPV1. To test this hypothesis, we firstly investigated the antinociceptive effects of PCW in TRPV1 wild-type and TRPV1^−/−^ mice. Our results showed that the antinociception of PCW was reduced in TRPV1^−/−^ mice compared to TRPV1^+/+^ group in inflammatory conditions, indicating TRPV1 was involved in antinociceptive process of PCW. Next, PCW antinociception was also explored in capsaicin tests in vivo and in vitro. Our data showed that PCW could significantly reduce capsaicin-induced nociceptive response, mechanical and heat hypersensitivities in mice. Since activation of the opioid receptors reduces TRPs-mediated cellular and/or behavioural responses [37–40], the antinociceptive effects of PCW were further examined on capsaicin-induced nociception with pretreatment of nor-binaltorphimine. Our findings demonstrated that PCW still manifested inhibitory effects on capsaicin-induced nociception with nor-BIN pretreatment, which further pointed to the connection of TRPV1 in PCW antinociception. Additionally, calcium image analysis confirmed the direct inhibitory action of PCW on TRPV1 ion channel. Taken together, these findings indicated that PCW may effectively inhibit TRPV1 activity, which were consistent with our recent report that Wu-Tou decotion, with the main component of PCW, could inhibit capsaicin-induced nociception in mice [41].

Throughout the present study, the antinociceptive effect of PCW was not susceptible to tolerance and the active dose of PCW 1.35 g/kg did not cause motor impairment, hyperthermia. Furthermore, the prolonged treatment of PCW (0.34–1.35 g/kg) did not cause significant change in body weight and gastrointestinal ulcers of mice. Therefore, it is likely that PCW selectively exerts its antinociceptive effect in inflammatory conditions. Current analgesics aim to modulate pain transduction and transmission in neurons has limited success in pain relieve [1], and targeting both the neuronal and nonneuronal mechanisms and excessive neuroinflammation have attracted considerable attention for better chronic pain treatments [1, 42]. Considering the main mechanisms of neuroinflammation and new notions of multi-targets intervention for chronic pain [1, 42], it is reasonable to believe that PCW might have better features than other known anagisics.

## Conclusions

Our data offered convincing evidence that PCW has potent antinociceptive effect in chronic inflammation conditions by attenuating mechanical and heat hypersensitivity without obvious side effect. These effects may result from the stimulation of κ-opioid receptor by increased dynorphin release in the lumber spinal cord and the inhibition of TRPV1 ion channel. Collectively, these findings confirm and add new information about antinociceptive properties of PCW, it also indicates that PCW might be a potential agent for the management of chronic inflammatory pain.

## Abbreviations

PCW: processed Chuanwu
NSAIDs: non-steroidal anti-inflammatory drugs
CFA: complete Freund’s Adjuvant
nor-BNI: nor-binaltorphimine
κ: kappa
TRPV1: transient receptor potential vanilloid type-1 ion channel
TRP: transient receptor potential
DRG: dorsal root ganglias
HEK293 cells: Human embryonic kidney cells
CCI: chronic constriction injury
i.t.: intraspinal injection
WT: wild-type
PWT: paw withdrawal threshold
PWL: paw withdrawal latency
MTT: 3-(4,5-Dimethylthiazol-2-yl)-2,5-diphenyltetrazolium bromide
